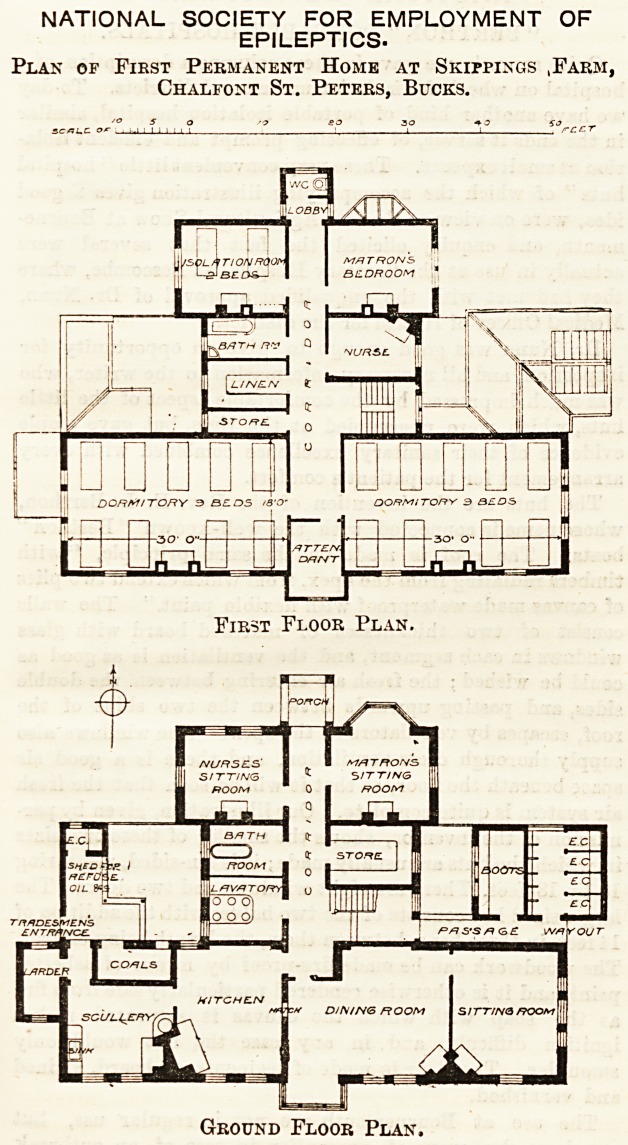# Hospital Construction

**Published:** 1895-07-27

**Authors:** 


### The Institutional Workshop.

### HOSPITAL CONSTRUCTION.

### NATIONAL SOCIETY FOR THE EMPLOYMENT OF EPILEPTICS.

The plans we publish to-day are those of the first permanent home for patients erected by the society. The colony was established in 1894 at Skipping's Farm, Chalfont St. Peters, Bucks, where towards the end of that year an iron building was opened for the

NATIONAL SOCIETY FOR EMPLOYMENT OF EPILEPTICS.

Plan of First Permanent Home at Skippings Farm, Chalfont St. Peters, Bucks.

First Floor Plan.

Ground Floor Plan.

reception of twenty male patients. The foundation-stone of the first permanent home was laid on November 14th, 1894, by Mr. Passmore Edwards. The building forms part of a general scheme for a group of homes, with a recreation hall to be erected hereafter, as funds permit.

The accommodation provided by the home is for eighteen patients, or colonists as they are called. On the ground floor is a large dining-room, with a sitting-room adjoining. These, with a bath-room, lavatory, boot cleaning-room, and closets, comprise the accommodation for the colonists. The administrative offices include sitting-rooms for the matron and nurses, store-room, and kitchen offices.

On the first floor are two dormitories for nine men each, with an attendants' room between having access to each dormitory, an isolation-room for two beds, matron's bed-room, nurses' bed-room, bath-room, linen-room, and w.c.

On the floor above is a large bed-room for two servants, and a cistern-room.

Both in planning and in construction the utmost economy had to be observed. The exterior is faced up to the first floor level with local red bricks, above which the walls are rough cast. The roofs are covered with red tiles. As the contract sum (exclusive of drainage) was £1,560 it will be seen that no unnecessary expense has been incurred. The plans were prepared by and the building erected under the supervision of Messrs. Young and Hall, Mr. Darlington, of Amersham, being the contractor.

## Figures and Tables

**Figure f1:**